# Two case reports of coronary spastic angina accompanied by the menstrual cycle

**DOI:** 10.1093/ehjcr/ytae381

**Published:** 2024-07-30

**Authors:** Rie Aoyama, Hironao Sudo, Shinichi Okino, Shigeru Fukuzawa

**Affiliations:** Department of Cardiology, Heart and Vascular Institute, Funabashi Municipal Medical Center, 1-21-1 Kanasugi, Chiba 273-8588, Japan; Department of Cardiology, Heart and Vascular Institute, Funabashi Municipal Medical Center, 1-21-1 Kanasugi, Chiba 273-8588, Japan; Department of Cardiology, Heart and Vascular Institute, Funabashi Municipal Medical Center, 1-21-1 Kanasugi, Chiba 273-8588, Japan; Department of Cardiology, Heart and Vascular Institute, Funabashi Municipal Medical Center, 1-21-1 Kanasugi, Chiba 273-8588, Japan

**Keywords:** Case series, Coronary spastic angina, Menstrual cycle, Estrogen, Acetylcholine stress test, Case reports

## Abstract

**Background:**

Coronary spastic angina (CSA) in premenopausal women is not frequent but has also been suggested to be associated with oestrogen decline during the menstrual cycle and sometimes becomes refractory and difficult to control. We experienced two premenopausal women with CSA that showed the involvement of the menstrual cycle.

**Case summary:**

Case 1: 41-year-old-woman had ST-segment elevation and chest pain during urosepsis, just 2 days after the onset of menstruation. The acetylcholine stress test was performed according to the menstrual cycle, and multiple coronary spasms were induced. Case 2: 40-year-old-woman had refractory chest pain as a symptom of premenstrual syndrome (PMS). Coronary angiography on drugs at the maximum dose revealed spontaneous multiple coronary spasms. Blood levels of oestrogen were normal, suggesting that hormonal change may be involved, and the introduction of low-dose pills made free from angina and the reduction of drug dose.

**Discussion:**

In premenopausal female angina pectoris, oestrogen may play a role; it is important to ask about the menstrual cycle and history of PMS. Besides, the timing of catheterization in premenopausal women with suspected CSA should be considered. Low-dose pills may be effective in some cases, and active medical collaboration with other departments such as gynaecology is desirable.

**Learning points**

- Coronary spastic angina (CSA) in premenopausal women is not frequent but has also been suggested to be associated with oestrogen decline during the menstrual cycle.
- The timing of catheterization in premenopausal women with suspected CSA should be considered.
- Low-dose pills may be effective in some cases, and active medical collaboration with other departments such as gynaecology is desirable.

## Introduction

In premenopausal women, ischaemic heart disease (IHD) is rare, but coronary spastic angina (CSA) and spontaneous coronary artery dissection (SCAD) are occasionally experienced in daily clinical practice. However, the symptoms and recurrence of these diseases often make it difficult to intervene clinically. Coronary spastic angina in premenopausal women is not frequent but has also been suggested to be associated with oestrogen decline during the menstrual cycle and sometimes becomes refractory and difficult to control. We hereby report two premenopausal women with CSA who showed the involvement of the menstrual cycle.

## Summary figure

**Table:** 

	Patient 1	Patient 2
Age	41 years old	40 years old
Preceding risk factor	Smoking, acute pyelonephritis	None
ECG change	ST elevation in II, III, and aVf	Negative T-wave in V1-V4 inductions
Day of attack	The second day of menstruation	A few days before the onset of menstruation
Coronary angiography (CAG)	Normal coronary	Normal coronary
Induction of coronary spasms by the acetylcholine (ACh) Stress test	RCA and LAD	LAD and LCX (not performed in RCA)
Medication before diagnosis	Diltiazem 100 mg and nicorandil 15 mg	Nifedipine 20 mg, isosorbide mononitrate 40 mg, and nicorandil 15 mg
Maximum dose medication	Diltiazem 100 mg and nicorandil 15 mg	Nifedipine 20 mg, diltiazem 400 mg, isosorbide mononitrate 40 mg, nicorandil 15 mg, and tocopherol nicotinate 900 mg
Final medication	Diltiazem 100 mg	Low-dose pills and Diltiazem 100 mg

## Case Report

### Patient 1

A 41-year-old-woman with no specific history of medical history or medications was admitted to a hospital for acute pyelonephritis and was started on antibiotics therapy (meropenem at a dose of 0.5 g per 12 h). She had smoking as a coronary risk factor. Her menstrual cycle was typically a 30-day cycle, and her menses lasted 7 days.

**Figure 1:**
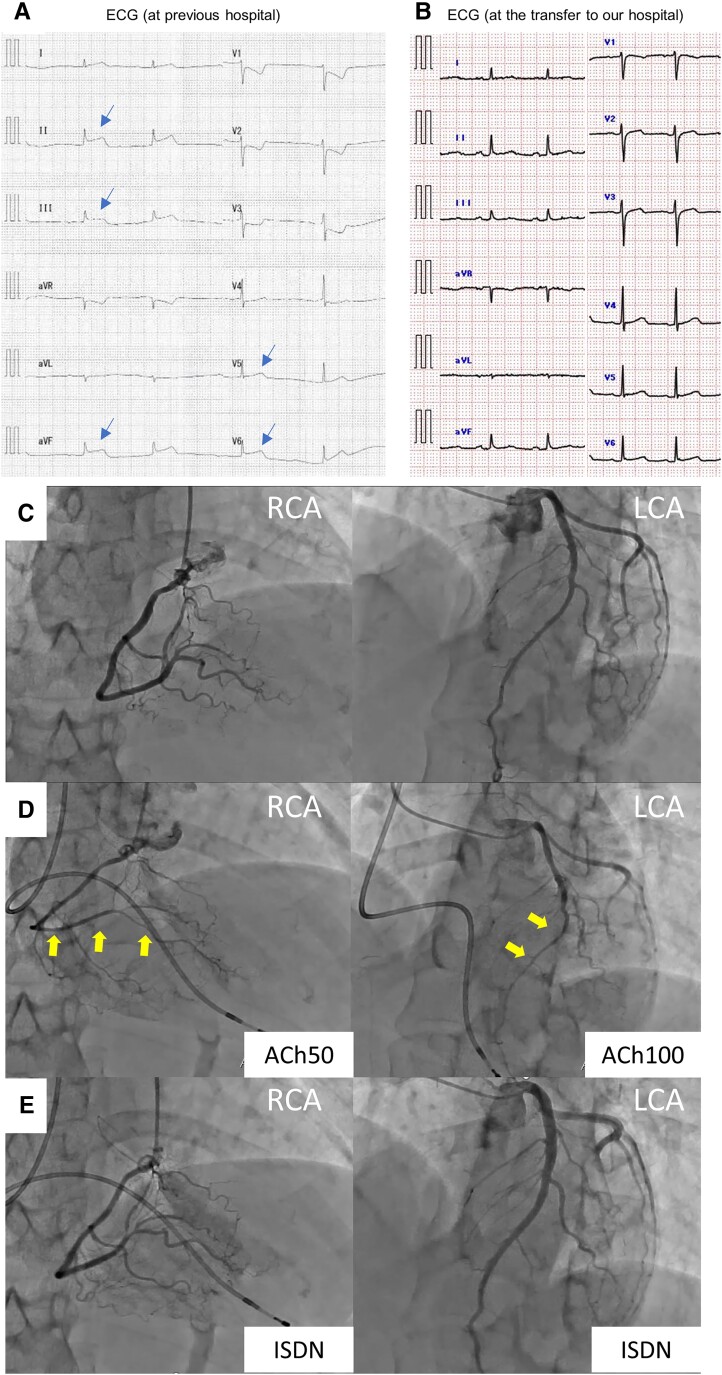
(*A*) Electrocardiogram at previous hospital demonstrating junctional rhythm and ST-segment elevation in leads Ⅱ, Ⅲ, aVF, and V5–6 (blue arrows). (*B*) Electrocardiogram at the time of transfer to our hospital demonstrating sinus rhythm and no ST-T changes. (*C*) Coronary angiography demonstrating no stenosis or occlusion in right coronary artery and left coronary artery. (*D*) Coronary angiography demonstrating coronary spasms induced in right coronary artery and left anterior descending coronary artery at 50 μg and 100 μg of acetylcholine, respectively (arrows). (*E*) Coronary angiography showing improvement of coronary spasm of RCA and left anterior descending coronary artery via intra-coronary administration of isosorbide dinitrate and nicorandil.

At 21 o’clock on Day 2, she suddenly had chest pain, and transient non-sustained ventricular tachycardia and complete atrioventricular block were observed (*Figure 1A*). That day was the second day of menstruation. She also had ST-segment elevation in II, III, aVf, and V5–6, and acute coronary syndrome was suspected. So, she was transferred to our hospital. When she was transferred to our hospital, she was in sinus rhythm and her ST-T changes had improved (*Figure 1B*). However, her blood pressure was 78/50 mmHg and her pulse rate was 91 b.p.m., and she was in a state of shock requiring noradrenaline (0.47 μg/kg/min) support. The results of a blood examination were as follows: white blood cell count (WBC) 10 600/μL; haemoglobin (Hb) 11.0 g/dL; platelet count (PLT) 4.6 × 103/μL; C-reactive protein 11.42 mg/dL; troponin-I 3.536 ng/mL (normal range, <0.06 ng/mL). Transthoracic echocardiography showed no wall motion abnormalities or significant valvular disease. After transfer to our hospital, antibiotics therapy was continued and intravenous nicorandil was started. No chest pain or no arrhythmia was observed. Pyelonephritis improved with the administration of antibiotics, and vital signs stabilized. Because of the transient ST-segment elevation and the fact that she was a young woman, the possibility of CSA associated with severe infection might be high, and we started a calcium channel blocker (CCB; diltiazem 100 mg). With the administration of nicorandil and CCB, she had a good clinical course without elevation of myocardial deviation enzymes and chest pain. As a young patient, it was necessary to determine whether CSA was transient and associated with infection or whether continued oral medication would be necessary.

Suspecting CSA in the acute phase of infection, we decided to perform a coronary angiogram (CAG) and an acetylcholine (ACh) stress test following her menstrual cycle. Two days before the onset of the next menstruation, CAG was performed under CCB discontinuation. No significant stenosis was observed on CAG (*Figure 1C*), and total occlusions due to coronary spasms were induced in the right coronary artery (RCA) and the distal part of the left anterior descending coronary artery (LAD) at 50 μg and 100 μg of ACh, respectively (*Figure 1D*). During the ACh stress test, a temporary pacemaker was inserted due to the possibility of bradycardia and the possibility that the stress may cause a strong spasm that is difficult to release. In this case, she had a transient complete atrioventricular block, and the pacemaker backed up. These stenoses of RCA and LAD were improved via intra-coronary administration of isosorbide dinitrate (ISDN) and nicorandil (*Figure 1E*). We diagnosed her with latent CSA, but this was her first attack and the severe infection might trigger CSA. So, the administration of CCB was continued, along with providing smoking cessation guidance. With smoking cessation and continued oral CCB use, she has remained stable with no recurrence of CSA during 2 years of outpatient follow-up.

### Patient 2

A 40-year-old-woman with no significant medical history, medication use, or cardiovascular risk factors had chest pain just a few days before the onset of menstruation. Despite drug therapy, she had refractory chest pain. Her menstrual cycle was typically a 28-day cycle, and her menses lasted 6 days.

**Figure 2:**
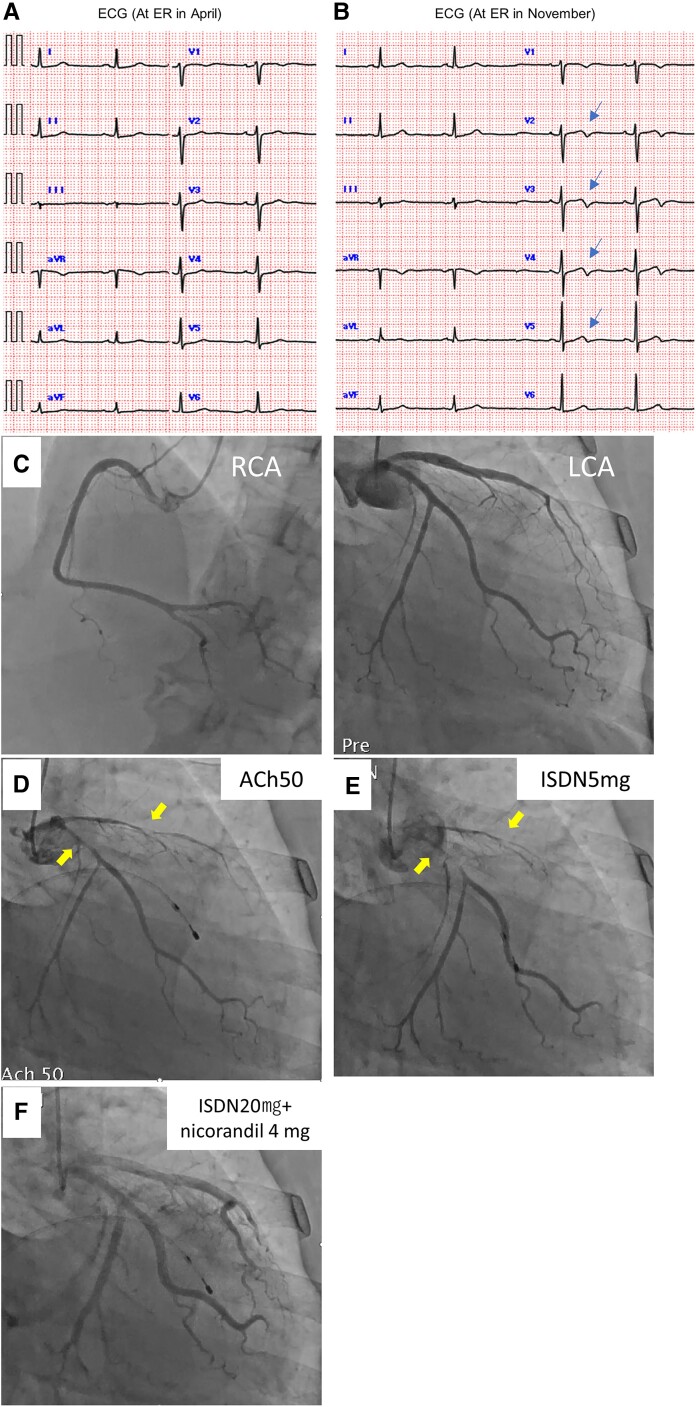
(*A*) Electrocardiogram in April demonstrating sinus rhythm and no ST-T changes. (*B*) Electrocardiogram in October demonstrating negative T-wave in leads V2–V5 (blue arrows). (*C*) Coronary angiography demonstrating no stenosis or occlusion in the right and left coronary arteries. (*D*) Coronary angiography demonstrating coronary spasms induced in the left anterior ascending coronary (LAD) and the left circumflex coronary artery (LCX) at 50 μg of acetylcholine (arrows). (*E*) Coronary angiography showing that coronary injection of ISDN 5 mg did not improve coronary spasm (arrows). (*F*) Coronary angiography showing improvement of coronary spasm of LAD and LCX via intra-coronary administration of isosorbide dinitrate 20 mg and nicorandil 4 mg.

She visited our emergency room because of resting chest pain that did not improve with nitroglycerin. Since there was no ECG change (*Figure 2A*) and no elevation of myocardial enzymes and CCTA showed no significant stenosis, CSA was suspected and CCB (nifedipine 20 mg) and ISMN (isosorbide mononitrate 40 mg) were started in April. Every month, she had chest pain just a few days before the onset of menstruation, but her anginal attacks were not accompanied by ECG changes or elevated myocardial deviation enzymes during the course.

She had strong chest pain and came to our emergency room in October. Her ECG showed negative T-wave in V1–V4 inductions (*Figure 2B*), but there was no elevation of myocardial deviation enzymes. The results of a blood examination were as follows: Hb 11.0 g/dL; LDL-C 103 mg/dL; troponin-I < 0.03 ng/mL; estradiol 62.3 pg/mL (normal range of the follicle period, 25–85 pg/mL). Transthoracic echocardiography showed no wall motion abnormalities or significant valvular disease. So, nicorandil 15 mg was added. She was poorly controlled on medication and agreed to undergo invasive testing to confirm the diagnosis and determine drug efficacy. Two days before the onset of menstruation in November, she was admitted to the hospital for CAG.

Coronary angiography was performed under medication (nifedipine 20 mg, isosorbide mononitrate 40 mg, nicorandil 15 mg) because her attack occurred even with medication. No significant stenosis was observed on CAG (*Figure 2C*), but 50 µg of ACh induced total or diffuse occlusions due to coronary spasm of left coronary artery (LCA; *Figure 2D*), which was not relieved by coronary injection of ISDN 5 mg (*Figure 2E*). Another coronary injection of isosorbide nitrate 15 mg and nicorandil 4 mg relieved multiple coronary spasms (*Figure 2F*). The ACh test to RCA was not performed because ST-segment was elevated during the ACh test to LCA. She was diagnosed with severe CSA because coronary spasm was induced even under drug administration and was not relieved by repeated administration of coronary dilators.

Diltiazem 400 mg and tocopherol nicotinate 900 mg were added because of insufficient control of the attack, but chest symptoms did not improve. Since her symptoms coincided with premenstrual periods, she was considered to have CSA associated with hormonal changes, and low-dose pills (combined oestrogen–progestin hormone contraception therapy with drospirenone and ethinylestradiol betadex) were introduced by the gynaecologist. The symptoms then became milder, and the oral dose was reduced to only 100 mg of diltiazem. She has been in good clinical course without the use of nitroglycerin.

The presence of attack as a symptom of PMS with the menstrual cycle suggested the involvement of sex hormones. Her blood levels of oestrogen were normal, suggesting that hormonal change may be involved, and the introduction of low-dose pills made free from angina and the reduction of drug dose. About 3 years have passed without chest pain attacks requiring the use of nitroglycerin tonics with continued use of CCB and low-dose pills.

## Discussion

There are gender differences in ischaemic heart disease. In men, coronary artery lesions are predominantly caused by atherosclerotic plaque, while in premenopausal women, atherosclerotic lesions are rare, and coronary spasm is the predominant complication.^1–3^ Coronary spastic angina due to epicardial coronary artery spasm is common in Japanese, especially in premenopausal women.^4–9^ Although the long-term prognosis of CSA is good and it rarely leads to sudden death and other fatal outcomes, we should understand the pathology of CSA and provide appropriate interventions because it worsens with the menstrual cycle and worsens their quality of life.

**Figure 3:**
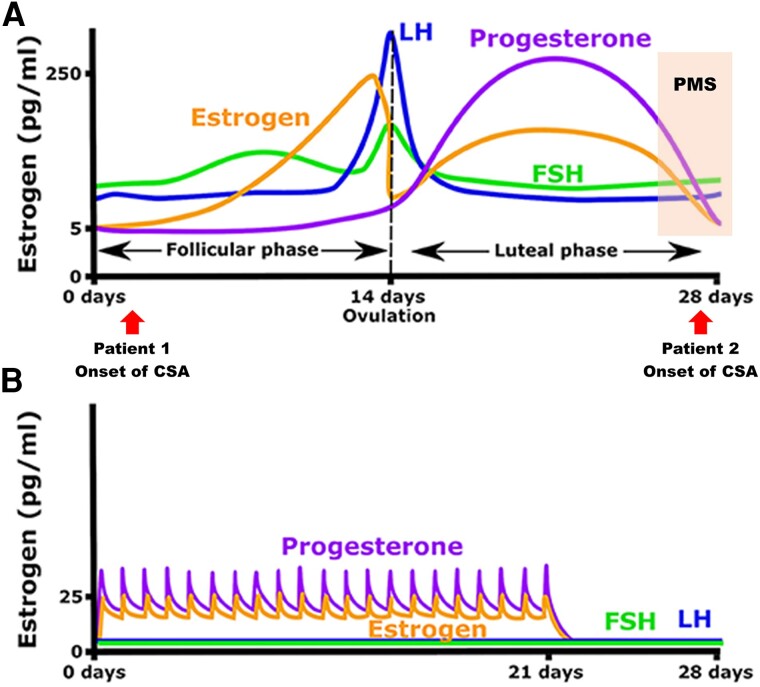
(*A*) Hormonal fluctuation during a normal menstrual cycle and premenstrual syndrome. The onset of coronary spastic angina attack in Patient 1 and Patient 2. (*B*) Hormonal fluctuation while combined oestrogen–progestin hormone contraception therapy.

Angina attacks in premenopausal women with CSA are closely related to endogenous oestrogen within the menstrual cycle.^10,11^ The frequency of attacks increases from the late luteal phase to the menstrual phase, when the oestrogen level and response of endothelium-dependent vasodilatation decrease, and decreases during the follicular phase when the oestrogen level and response of the endothelium-dependent diastolic vasodilatation increase.^12^ Premenstrual syndrome (PMS) is a mental or physical symptom that lasts for 3–10 days before menstruation and disappears with the onset of menstruation. The cause of PMS is thought to be a rapid decrease in oestrogen and progesterone secretion. Coronary spastic angina associated with the menstrual cycle often occurs as a symptom of PMS shows increased attacks during the menstrual phase and may be related to the variability of oestrogen concentrations rather than absolute oestrogen blood concentrations (*Figure 3A*).^13–15^

Therefore, when CSA is suspected in premenopausal women, the timing of cardiac catheterization should be considered. If the ACh stress test^16^ is performed during the follicular phase, when the endogenous oestrogen level is high, it may not induce coronary spasm. We experienced two cases that showed the onset of CSA as part of the PMS symptoms. In Case 1, severe infection and smoking habit might induce a latent coronary spasm. And partly due to problems with the menstrual cycle, therefore, the ACh stress test for definitive diagnosis was performed in the luteal phase because the ACh stress test in the luteal phase may be more likely to be positive, considering the timing of the ACh stress test might be helpful in the diagnosis and the treatment of CSA in premenopausal women.

In Case 2, CSA attacks were limited to PMS, and the chest symptoms disappeared as soon as menstruation started (*Figure 3A*). In premenopausal healthy women, the response of the endothelium-dependent vasodilatation fluctuates with fluctuations in endogenous oestrogen levels during the menstrual cycle.^17^ Therefore, premenopausal women do not always benefit from the anti-atherosclerotic effects of oestrogen. In particular, premenopausal women with risk factors for atherosclerosis, such as diabetes mellitus and familial dyslipidaemia, may be more susceptible to acute coronary syndromes from the luteal phase to the menstrual phase. Some cases of refractory CSA resistant to standard drug therapy require attention. In Case 2, despite the administration of drugs at their maximal doses, angina episodes occurred repeatedly. It has been suggested that a decrease in oestrogen levels during certain phases of the menstrual cycle may trigger coronary spasms. In some cases, hormone therapy such as low-dose pills is effective (*Figure 3B*).^18,19^

Providing appropriate treatment based on careful history-taking and understanding its pathophysiology is important. In premenopausal female angina pectoris, oestrogen may play a role; it is important to ask about the menstrual cycle and history of PMS. Low-dose pills may be effective in some cases, and active medical collaboration with a gynaecology department is desirable.

## Data Availability

The data underlying this article will be shared on reasonable request to the corresponding author.

## Lead author biography

Rie Aoyama is a physician in the Department of Cardiology of Funabashi Municipal Medical Center and an interventional cardiologist. Her clinical interests include PCI, coronary imaging, TAVR, and interventional approach for hypertrophic cardiomyopathy. She graduated from Nippon Medical Graduate School, PhD in coronary imaging and interventional approach for hypertrophic cardiomyopathy. Her research interests include coronary intervention, heart failure, and exploring sex-based differences in cardiovascular disease.

**Consent:** Written consents for submission and publication of this case report including images were obtained from the patients in line with the COPE guidelines.

**Funding:** None declared.
